# Factors associated with engagement in HIV care for young people living with perinatally acquired HIV in England: An exploratory observational cohort study

**DOI:** 10.1371/journal.pone.0302601

**Published:** 2024-05-24

**Authors:** Marthe Le Prevost, Ali Judd, Siobhan Crichton, Caroline Foster, Alasdair Bamford, Deborah Ford, on behalf of the Adolescents; Adults Living with Perinatal HIV (AALPHI) Steering Committee

**Affiliations:** 1 MRC Clinical Trials Unit at University College London, London, United Kingdom; 2 Imperial College Healthcare NHS Trust, London, United Kingdom; 3 Great Ormond Street Hospital for Children NHS Foundation Trust, London, United Kingdom; Boston University School of Medicine, UNITED STATES

## Abstract

Identifying which young people living with perinatally acquired HIV (PHIV) are less likely to engage in care is crucial to allow targeted interventions to support them to attend clinic. We adapted an existing Engagement in Care (EIC) algorithm for adults with HIV in England, for use in young people. We applied it to data from young people with PHIV in the Adolescents and Adults Living with Perinatal HIV (AALPHI) cohort. The algorithm predicts the timing of the next scheduled clinic visit, within 1–6 months of current visit, based on routine clinical data. Follow-up was 12-months from AALPHI baseline interview. Each person-month was classified as engaged in care or not. Logistic regression models (allowing for clustered data) were used to explore baseline characteristics associated with being engaged in care, adjusting for *a priori* variables (time from interview, sex, age, ethnicity, country of birth). Potential characteristics were across 7 domains: sociodemographic; risk behaviour practices; mental health; cognition; clinic setting; HIV management and experience; and HIV clinical markers. Of 316 young people, 187(59%) were female, 271(86%) of black ethnicity and 184(58%) born abroad. At baseline, median [IQR] age was 17[15–18] years, and 202(69%) had viral load ≤50 copies/ml(c/mL). 87% of 3,585 person-months were classified as engaged in care. Characteristics independently associated with poorer odds of being engaged in care were: Asian/mixed/other ethnicity, vs. black ethnicity (OR 0.44, 95% CI 0.25, 0.78, p = 0.02); ever self-harmed, vs. not (OR 0.55, 95% CI 0.32, 0.95, p = 0.03); on antiretroviral therapy (ART) and self-assessed bad/not so good adherence (OR 0.46, 95% CI 0.25, 0.84) or not on ART (OR 0.64, 95% CI 0.64, 1.21) vs. on ART and good/excellent adherence (p = 0.04)); baseline VL>50c/mL, vs VL≤50c/mL (OR 0.47, 95% CI 0.30, 0.75, p = 0.002). These characteristics can help identify individuals requiring enhanced support to maintain service engagement.

## Introduction

There are many familial, social and developmental complexities growing up with perinatally acquired HIV (PHIV), which makes ongoing self-management a chronic condition through adolescence and adulthood challenging [1–8]. This is reflected in the often poorer health outcomes of young people living with PHIV compared to other age groups [9, 10].

Engagement in care (EIC) has been associated with improved patient outcomes and is a focus of global and national targets [11–14]. EIC is especially important in young people with PHIV as they are a particularly vulnerable group. However, few studies have measured EIC in children and young people with PHIV in Europe, and none have looked at characteristics associated with EIC, which could help identify young people at increased odds of disengagement. Furthermore, most studies of EIC in people living with HIV use a simplistic definition of EIC based on the number of clinic visits per year.

In a previous paper [15], we report how we adapted an existing EIC algorithm for adults with HIV in England [16], for use in young people and applied it to young people living with PHIV in the Adolescent and Adults Living with Perinatal HIV (AALPHI) cohort in 2013–2015. AALPHI was a prospective observational study evaluating the impact of HIV infection and ART exposure in young people in England [17, 18]. Here we build on that papers results by exploring a broad range of potential characteristics of EIC. The aim was that these characteristics could be useful to clinicians to identify and provide additional interventions for young people living with PHIV who are more likely to disengage from HIV care.

## Materials and methods

### EIC outcome measure

The EIC algorithm used for this analysis was adapted from an existing adult algorithm [16] which predicts when the next clinic visit will be scheduled (within 1–6 months) following the current visit, based on routinely collected clinical data (e.g., CD4 and viral load). The algorithm was adapted for use in young people living with PHIV in consultation with paediatric and adolescent HIV consultants from the UK [15]. Three flowcharts were developed for young people based on a person’s clinical data at the current clinic visit (see flowcharts in S1–S3 Figs). Flowcharts were used to predict visits over a 12 month period, compared to appointment attendances, to measure whether young people were engaged in care in each month (patient attended clinic early, within the month or not yet due for clinic visit) or not engaged in care (patient ≥15 days overdue a clinic visit) [15]. In any given month a patient was in care if they had a clinic visit or their next visit was not yet due. A patient was considered out of care if they did not attend a visit and their visit was at least 15 days overdue. When a patient attended following a period of disengagement (one or more months), they were considered to be reengaged in care in the month the visit occurred.

### The Adolescents and Adults Living with Perinatal HIV (AALPHI) cohort

Detailed methods used in AALPHI have been described elsewhere [17, 18]. Briefly, AALPHI commenced in 2012 and recruited two groups of young people, those with PHIV, and a comparison group who were HIV negative but HIV-affected. Both groups were recruited from NHS clinics and voluntary sector organisations across England. Young people living with PHIV were: aged 13–21 years, previously or currently receiving paediatric care in England, able to give informed consent/assent and aware of their HIV diagnosis for at least 6 months. Ethical approval was obtained from Leicester Research Ethics Committee. All AALPHI participants with PHIV were in the Collaborative HIV Paediatric Study (CHIPS) cohort, a national surveillance cohort of all children in HIV care in the UK [19]. Routine clinical data from CHIPS and data from the baseline AALPHI interview were considered as potential characteristics associated with EIC. Data were last accessed on 21/08/2023.

### Potential characteristic associated with EIC

Published papers of factors known to be associated with EIC were reviewed and expert opinion sought, to select potential characteristics for this analysis. In addition, characteristics that may affect recognised factors associated with EIC were also considered (see S1 File for a list of all variables in the AALPHI and CHIPS cohorts, and S2 File for the rational for all included variables). For example, mental health has been found to be associated with poorer EIC [20] therefore variables associated with mental health status (such as exclusion from school and death of parents) were included. The conceptual framework used for this analysis was developed from the work of Harberer and Mellin [21] on adherence to ART in young people living with PHIV. They recommended that due to the complex challenges of long-term adherence, four categories, or domains, need to be taken into consideration when planning interventions; the child; caregiver(s) and family; the ART regimen; and society and culture [21]. Our study benefited from measurement of a wide range of potential exposure variables, far beyond the more limited core clinical HIV factors included in many analyses. We extended Harberer and Mellin’s domains by grouping these variables into 7 domains (Table 1) and adjusted for *a priori* variables. *A priori* variables were time from interview, sex, age, ethnicity, country of birth. Management of missing data are described elsewhere [15].

**Table 1:** A priori and baseline characteristics considered in EIC models, grouped by 7 domains.

Domain	Variables
*A priori* domain	Months since AALPHI baseline interview, sex, age, ethnicity, born outside of the UK/Ireland
Sociodemographic domain	Education/employment status, ever excluded from school, parent vital status, fostered/adopted, number of main carers, live with parents/carers, parent/carer employment status, main language spoken at home, IDACI deprivation score
Risk behaviour practices domain	Ever smoked cigarettes, use of alcohol and recreational drugs, ever had sex (anal or vaginal), age at first sex, use of condoms
Mental health domain	Perceptions about HIV, ever self-harmed, ever felt life not worth living, major life events, quality of life, self-esteem, anxiety, depression
Cognition domain	NPZ-6 score^1^
Clinic setting domain	Clinic location, clinic type, distance from home to clinic, travel time from home to clinic
HIV management and experience domain	Age at HIV naming, how many people participants have told about their HIV, number of doses missed in last three days, self-assessment of ART adherence
HIV clinical markers domain	CDC C stage, CD4 nadir, current CD4, current VL (≤50c/mL/>50 c/mL^2^), time on ART, on efavirenz^3^, treatment interruption in last 2 years (gap ≥30 days)

^1^NPZ-6 score: A cognitive composite of executive functions (neurophysiological scores (NPZ score)). Mean score calculated using manufacturer normative data for six domains: executive function; speed of information processing; attention/concentration, learning, memory and fine motor skills.

^2^ Categorised continuous variable

^3^ Included due to potential link to depression

### Statistical analysis

The outcome for all models was in care vs out of care in each month. Multivariable logistic models were fitted with generalised estimating equations to account for multiple months per participant. Stepwise backward selection (p<0.15) was used to identify characteristics in 7 domain specific models adjusting for *a priori* variables. These domain-specific characteristics were then fitted in a combined multivariable model, adjusting for *a priori* variables, using stepwise backward selection (p<0.05).

Three sensitivity analyses were conducted. First, the p-value for retention in the combined model was relaxed to p<0.01, as that may be more appropriate with small sample sizes. Second, an alternative baseline date, three months after the AALPHI interview date, was used, as there could be a bias from using the AALPHI interview date as the start date of follow-up time. Over half (57%, n = 175/306) of the AALPHI cohort did not have a clinic visit on the same day as their AALPHI interview and consequently did not have clinical data available on this day. Therefore, these participants could potentially start the follow-up period classified as ‘out of care’, while those with a clinic visit on the same day as their AALPHI interview date were always considered ‘in care’ at baseline. Third, the maximum interval for predicted time to next scheduled visit was reduced from 6 months to 4 months. Appointment visits predicted by the EIC flowcharts at 6 months were the visits that participants most frequently attended early, raising questions about whether this gap was longer than used in clinical practice (see S3 File for further details). All analyses were conducted in STATA version 15 [22].

## Results

Of 316 young people living with PHIV included in this analysis (Table 2, column a), just under two thirds were female (59%) and median age was 17 years [IQR 15–18]. Most young people were of black ethnicity (86%), and born outside the UK/Ireland (58%). At baseline, the majority of young people were on ART (88%), two thirds (69%) had a suppressed viral load (≤50 c/mL) and the median CD4 count was 597 cells/μL [427, 791]. In total there were 3,585 person-months of follow-up, with 87% (3,126/3,585) of months fulfilling the definition of engaged in care.

**Table 2:** Characteristics associated with EIC in domain-specific and combined multivariable models.

Domain, variable and category (all baseline)	a. Baseline characteristics^1^ n (%) [IQR] (total n = 316)	b. Domain-specific models adjusted for *a priori* variables^2^		c. Combined model^2^	
		OR (95% CI)	p-value	OR (95% CI)	p-value
***A priori* domain**					
Follow-up time, per month later				0.97 (0.93, 1.02)	0.24
Sex					
Male	129 (41)			1	-
Female	187 (59)			1.29 (0.86, 1.94)	0.22
Entry age, per year increase	17 [15,18]			0.94 (0.87, 1.02)	0.15
Ethnicity					
Black	271 (86)			1	
Asian/mixed/other	34 (11)			0.44 (0.25, 0.78)	
White	9 (3)			1.05 (0.29, 3.81)	0.02
Born outside the UK/Ireland					
No	132 (42)			1	-
Yes	184 (58)			0.86 (0.57, 1.30)	0.47
**Sociodemographic domain**					
Ever excluded from school					
No	259 (82)	1			
Yes	55 (18)	0.61 (0.38, 0.99)	0.04		
Main language spoken at home					
English/English and another language	307 (97)	1			
A language other than English	8 (3)	0.52 (0.24, 1.16)	0.11		
**Risk behaviour practices domain**					
Current alcohol amount (past year)					
No drinking	174 (59)	1			
Light drinking	92 (31)	0.64 (0.40, 1.01)			
Hazardous drinking	30 10)	0.57 (0.28, 1.19)	0.12		
Condom use					
Not sexually active	195 (68)	1			
Always use a condom	63 (22)	1.76 (0.95, 3.26)			
Do not always use a condom	30 (10)	0.80 (0.42, 1.54)	0.07		
**Mental health domain**					
Ever self-harmed					
No	265 (88)	1		1	
Yes	37 (12)	0.66 (0.38, 1.14)	0.14	0.55 (0.32, 0.95)	0.03
**Clinic setting domain**					
Clinic type					
Paediatric	234 (75)	1			
Adolescent	52 (16)	1.42 (0.67, 3.00)			
Adult/GUM clinic	27 (9)	3.88 (1.00, 15.10)	0.14		
Travel time to clinic, per 10 min increase	31 [19.46]	0.97 (0.94, 1.00)	0.08		
**HIV management and experience domain**					
ART adherence self-assessment					
On ART, adherence excellent or good	225 (77)	1		1	-
On ART, adherence not so good or bad	34 (12)	0.39 (0.23, 0.64)		0.46 (0.25, 0.84)	0.04
Not on ART	35 (12)	0.35 (0.21, 0.59)	<0.001	0.64 (0.34, 1.21)	
**HIV clinical markers domain**					
Viral load					
≤50c/mL	202 (69)	1		1	-
>50c/mL	89 (31)	0.33 (0.23, 0.48)	<0.001	0.47 (0.30, 0.75)	0.002

Abbreviations: IQR–Interquartile range

^1^Baseline characteristics are summarised among participants with complete data for each variable.

^2^Models were based on 306 participants (10 participants were dropped from the modelling stage due to missing data)

Table 2 shows characteristics associated with EIC from the domain-specific (column b) and combined models (column c). In the combined model, ethnicity, ever self-harmed, adherence self-assessment and viral load were independently associated with EIC. Young people were less likely to be engaged in care if they were of Asian/mixed/other ethnicity (OR 0.44, 95% CI 0.25, 0.78) compared with white or black ethnic groups (white OR 1.05, 95%CI 0.29, 3.81, reference black ethnicity, p = 0.02). They were less likely to be engaged in care if they had ever self-harmed (OR 0.55, 95% CI 0.32, 0.95) compared to those who had not self-harmed (p = 0.03). They were also less likely to be engaged in care if they were on ART and self-assessed their adherence as not so good or bad (OR 0.46, 95% CI 0.25, 0.84) or were not on ART (OR 0.64, 95% CI 0.34, 1.21) compared to those on ART who assessed their adherence as excellent or good (p = 0.04). Finally young people were less likely to be engaged in care if they had a baseline viral load >50c/mL (OR 0.47, 95% CI 0.30, 0.75) compared to ≤50c/mL (p = 0.002).

### Sensitivity analyses

Results in all three sensitivity analyses (using a significance level of 0.1, delayed start date and reduced maximum appointment interval from 6 to 4 months) were similar to the combined main model.

## Discussion

In this paper, we investigated the association between a broad range of characteristics and EIC in young people living with PHIV. To our knowledge this is the first analysis that examines characteristics associated with EIC in young people living with PHIV in the UK, and the first multi-clinic analysis in Europe. Using individual patient data from the AALPHI and CHIPS cohorts, we were able to measure EIC in a more nuanced way than many previous analyses [23–26] by taking into account participants’ clinical characteristics. Variables were grouped into eight domains, and four variables were found to be associated with EIC, each from a different domain. From the *a priori* domain, young people were less likely to be engaged in care if they were of Asian/mixed/other ethnicity compared to being of black or white ethnicity. From the mental health domain, participants had worse EIC if they reported having ever self-harmed. From the HIV management and experience domain participants were less likely to be engaged in care if they reported having not so good/bad adherence to ART, or if they were not on ART, compared to excellent/good adherence to ART. Finally from the “clinical markers” domain, participants had poorer EIC if they had a viral load >50c/mL compared to ≤50c/mL. Results of sensitivity analyses were reassuringly similar to the main combined model, with ethnicity, self-harm, adherence and viral load remaining associated with EIC across all three sensitivity analyses. In addition, there was evidence from all three sensitivity analyses of a potential effect of older age and worse EIC.

It is important to note that relatively few studies have looked at EIC in young people living with PHIV. Additionally the method of measuring EIC varies widely in the literature, and often most characteristics are from the *a priori* and HIV clinical markers domains, limiting direct comparisons. This paper used an adapted algorithm for EIC which was originally developed by Howarth *et al* [16] for adults with HIV in the UK, and results are consistent across the age groups. Howarth et al [16] demonstrated that age was also associated with EIC in univariable analyses, with the proportion of patients engaged in care being lowest in the 16–24 years age group (77% engaged in care, vs. 83% for 25–45 years and 87% for >45 years). However after adjustment for other factors the association was weaker (16–24 years p = 0.09, 25–45 years p = 0.008 vs >45 years). In terms of ethnicity, they also reported that people who identified as being in an ethnic group other than black African or white [16] were less likely to be engaged in care, similar to our findings. Such patients are minority ethnic groups in HIV clinics in the UK, and may have specific barriers to engaging in care. Howarth *et al* [16] also reported that people who were on ART were more likely to be engaged in care than people who were off ART, similar to our findings. They also found that patients with lower nadir CD4 counts were less likely to be engaged in care, and did not look at the effect of viral load [16]. Our model included both, and CD4 nadir was dropped in favour of viral load.

In addition to Howarth *et al*, [16] two other studies identified associations between age and EIC. Gray *et al* [23] in the USA, reported that young people living with PHIV aged 13–17 years had a higher EIC that 18–25 year olds. Similarly, Gebrezgi *et al* [27] reported that 13–17 year olds had better EIC than 18–20 year olds and 21–24 year olds. This is similar to the trend shown in our analysis with EIC declining as young people aged.

Other studies, where EIC was more simply defined, have also found associations between ethnicity and EIC. In Gray et al’s [23] analysis of young people living with PHIV across the USA (n = 11,747), people of Hispanic/Latino ethnic group were more likely to be engaged in care than people of white ethnicity. In contrast, in a cohort of young people aged 13–24 years with HIV in Florida (n = 2,872), Gebrezgi *et al* [27] in their cohort of young people with mainly horizontally acquired HIV reported that Hispanics were less likely to be engaged in care than people of non-Hispanic white ethnicity. These contradictory findings are perhaps unsurprising and difficult to compare. Ethnicity in the context of these studies, as well as our own, can be considered a measure of health disparities caused by structural inequalities and discrimination [28]. The complexity of these factors and the impact on engagement in care of young people living with HIV may differ across the different social and political contexts in the UK and US as well as between different groups of people.

In our analysis, self-harm was the only mental health factor, and adherence the only HIV management and experience factor, associated with EIC. Most other studies of EIC included only routinely collected sociodemographic and clinical factors in their analyses, and it may be challenging to enable honest reporting of self-harm and adherence by young people in clinic. A previous analysis from our group suggested that in the AALPHI cohort, self-esteem was strongly associated with self-harm, with each one point increase in the Rosenberg Self-Esteem Scale (higher score = better self-esteem) being associated with a 10% reduction in the odds of self-harm [29]. Similarly in the Avon Longitudinal Study of Parents and Children (ALSPAC) general population (non-HIV specific) study, concurrent depression was associated with a much increased risk of self-harm [30]. Any indication of mental health issues might thus be considered a flag for risk of disengagement with care.

### Limitations

This analysis has several limitations and possible sources of biases that could affect the findings. The EIC outcome was measured across one year for each participant, whilst the characteristics were measured at baseline (AALPHI interview). This approach was taken to avoid time dependent confounding, however variables including baseline viral load and adherence may have been influenced by previous EIC, meaning it is still not straightforward to disentangle cause and effect. Furthermore, a substantial proportion of participants were in care at baseline, meaning effects of baseline characteristics may have been diluted. In addition, given most participants were in care at baseline and most were on ART, young people in this analysis were a largely engaged cohort and we have therefore not captured those who were already lost to follow-up. However, young people were less likely to be engaged in care if they had unsuppressed virus, or if they self-reported poor adherence, or were off ART, which shows that many young people remained in care despite having problems taking their ART. For this analysis, variables were considered within domains and much of the selection took place prior to considering the full multivariable model including variables across these domains, meaning between-covariate associations may have been missed. In this analysis, we found that some participants attended their appointments early, however as we do not have reasons for visits we do not know the reasons for early attendance. There are a lack of tools specifically designed and validated in young people and compromises in tool selection were required to ensure the AALPHI interview was not too lengthy for the participants. Lastly, social desirability or misinterpretation/misunderstanding of question can impact answers given.

## Conclusion

In conclusion, the richness of the AALPHI dataset enabled us to explore the potential effect of a broad range of variables on EIC. Many clinics already use viral load as an indicator that young people may be disengaging with care. This analysis suggests that in addition to viral load, simple assessments of adherence and self-harm (or other mental health measures) may have added benefit in identifying young people most at need of targeted interventions to optimise clinic attendance.

## Supporting information

S1 Fig Group A flowchart—visits in young people living with PHIV on ART with viral load ≤50c/mL (n = 235). (DOCX)

S2 Fig Group B flowchart—visits in young people living PHIV on ART with viral load >50c/mL (n = 112). (DOCX)

S3 Fig Group C flowchart–visits in young people living with PHIV off ART (n = 35). (DOCX)

S1 File Variables in the AALPHI and CHIPS cohorts. (DOCX)

S2 File Variables included in the analysis and rationale for inclusion. (DOCX)

S3 File Early attendance for appointments. (DOCX)
