# Supplementary material for: Factors associated with engagement in HIV care for young people living with perinatally acquired HIV in England: An exploratory observational cohort study
Source: PLoS One. 2024 May 24;19(5):e0302601. doi: 10.1371/journal.pone.0302601 (PMC11125550; doi:10.1371/journal.pone.0302601)
Supplement: S1 Fig — (DOCX) [file pone.0302601.s001.docx]

# Group A Flowchart - visits in young people living with PHIV on ART with viral load ≤50c/mL (n=235)

**S1 Fig. Group A Flowchart - visits in young people living with PHIV on ART with viral load ≤50c/mL (n=235)**

#### ^1^ART=Antiretroviral therapy

#### ^2^ VL= viral load

#### ^3^Proportions given at the terminal nodes of decision trees

#### ^4^Regimen change=on continuous therapy but a component changed
