# Supplementary material for: Factors associated with engagement in HIV care for young people living with perinatally acquired HIV in England: An exploratory observational cohort study
Source: PLoS One. 2024 May 24;19(5):e0302601. doi: 10.1371/journal.pone.0302601 (PMC11125550; doi:10.1371/journal.pone.0302601)
Supplement: S2 Fig — (DOCX) [file pone.0302601.s002.docx]

**Group B Flowchart - visits in young people living PHIV on ART with viral load >50c/mL (n=112)**

**S2 Fig. Group B Flowchart - visits in young people living PHIV on ART with viral load >50c/mL (n=112)**

#### ^1^ART=Antiretroviral therapy

#### ^2^ VL= viral load

#### ^3^Proportions given at the terminal nodes of decision trees

#### ^4^Regimen change=on continuous therapy but a component changed

#### ^5^PI-protease inhibitor
