# Supplementary material for: Factors associated with engagement in HIV care for young people living with perinatally acquired HIV in England: An exploratory observational cohort study
Source: PLoS One. 2024 May 24;19(5):e0302601. doi: 10.1371/journal.pone.0302601 (PMC11125550; doi:10.1371/journal.pone.0302601)
Supplement: S3 Fig — (DOCX) [file pone.0302601.s003.docx]

**Group C Flowchart – visits in young people living with PHIV off ART (n=35)**

**S3 Fig. Group C Flowchart – visits in young people living with PHIV off ART (n=35)**

^1^ART=Antiretroviral therapy

^2^Proportions given at the terminal nodes of decision trees
