# Supplementary material for: Factors associated with engagement in HIV care for young people living with perinatally acquired HIV in England: An exploratory observational cohort study
Source: PLoS One. 2024 May 24;19(5):e0302601. doi: 10.1371/journal.pone.0302601 (PMC11125550; doi:10.1371/journal.pone.0302601)
Supplement: S1 File — (DOCX) [file pone.0302601.s004.docx]

**S1: List of variables in the AALPHI and CHIPS cohorts**

**S1 Table. Variables in the AALPHI and CHIPS cohorts**

| **AALPHI** | |
| --- | --- |
| **Category** | **Variables collected** |
| Demographics | - sex - age - ethnicity - country of birth - if born abroad age moved to UK |
| Social factors | *Young person’s information:*   - marital status - details of biological children (how many, information about developmental delay or physical problems) - education history (existing qualifications; receipt of educational maintenance allowance/bursary; years spent in education; ever received fixed/permanent exclusion; if not still in school, reason for leaving; awarded place at university, and if yes, university name and details - employment history (if in work, main work activity; details of current paid job; start date; description of main activities; history of all paid jobs; dates of unemployment) - accommodation/living circumstances (if live with parents/carers, how many adults/children in house and who they are; if live outside family home, who live with, and in the last family home, how many adults/children were there in house and who were they, type of home) - number of main carers; fostered/adopted - contact with social service/youth offenders/school educational support - language spoken at home - voluntary work history - time off on sick leave - parents’ employment status (employed/unemployed; if working, job classification; if unemployed, for how long, job classification of last job) - parents’ vital status (if died, age of death and cause of death) - parents’ country of residence - Income Deprivation Affecting Children Index (IDACI) residential deprivation score based on postcode |
| Medical history | - prematurity, birth weight - medical history (e.g. diabetes type I/II, hypertension, heart disease, rheumatoid arthritis) - non-ART medications (name, reason for medication, length of time taking medication) - bone fracture history (age, bone broken, how it happened) - family medical history (e.g. diabetes type I/II, stroke, hypertension, heart disease) - exercise (time categories per day in last month) |
| Quality of life and mental health | - quality of life (Pediatric Quality of Life Inventory (PedsQL^TM^)) - self-esteem (Rosenberg Self-Esteem Scale) - self-harm and suicidal ideation - anxiety and depression (Hospital Anxiety and Depression Scale) - psychology (mental health service contact/referrals) - life events (Avon Longitudinal Study of Parents and Children (ALSPAC) questionnaire from ALSPAC ‘Life of a 16+ Teenager’ questionnaire) |
| Alcohol and recreational drug use | - alcohol use (Alcohol Use Disorders Identification Test (AUDIT)) - smoking history - recreational drug use |
| Growth and sexual health | - feelings about body image - referral for anorexia/obesity - Tanner Staging self-assessment - females only: menstrual pattern/history (age at menarche; days between periods; date of last period; receipt of contraception pill to regulate periods) - HPV vaccine (location vaccine was offered/other locations young people would like to be offered vaccine; whether agreed, and if yes, how many vaccinations, vaccination type; reason decided to have/not have HPV vaccine) - HBV vaccination history (if yes, was course completed) - sexual health history (had anal/oral vaginal sex; if yes, age at first intercourse, no. partners in last 12 months, relationship with first/most recently person had sex with, no. of sexual partners, STI screening in last 12 months; ever had STI, and if yes, which STI; if no intercourse, reason) - contraception use (condom use; birth control methods; for females only, if using oral contraceptive, how many missed in last month, contraceptive injection details, emergency contraception use) - post-exposure prophylaxis (PEP) and pre-exposure prophylaxis (PrEP) - females only: plans to get pregnant - pregnancy history (if yes, date of last pregnancy test; currently pregnant; if yes, plan to have baby, due date; if no, termination booked; how many times pregnant; how many live births; if not live births, outcome (termination, miscarriage, ectopic, still birth), if live birth-birth history (age/gestation at delivery; child’s date of birth; hours in labour; type of birth; sex and weight of child; breastfed; HIV status)) |
| HIV naming | - age told HIV status - how found out (where told, how told/found out, by whom) - feelings about HIV (at time of HIV naming and currently) - talking about HIV (who talk to, who talk to most) - who knows (does everyone in the family know, how many people know, how many people told) |
| Adherence to ART | - if on ART, frequency of taking medicines - missed doses (doses especially difficult to take; reminders used; number of missed doses in last 3 days/month; self-assessment of adherence; reasons for any missed doses) - If not on ART, main reasons for stopping |
| Cognition | - executive function (Cogstate Groton Maze and Color Trials 2) - speed of information processing (Cogstate Detection Task and Color Trials 1) - attention/concentration (Cogstate Identification Task and WAIS-IV) - learning (Cogstate One Card Learning and Cosgstate International Shopping List) - memory (Cogstate International Shopping List Delayed and Cogstate One Back Task) - fine motor skills (Grooved Pegboard) |
| Samples | - ETDA plasma, SST serum, Lithium heparin plasma |
| Physical assessments | - blood pressure - height/weight measurements, hip/waist measurements - face changes |
| **CHIPS** | |
| **Category** | **Variables collected** |
| Demographics | - age - partial postcode |
| HIV related | - immune function (CD4 / CD8 / total lymphocytes) - HIV viral load - CDC C or B disease stage classification events |
| ART | - names of ART drugs - dose/formulation - date started/stopped - reasons for start/stop |
| ART adverse related events | - details of adverse events Grades ≥2 (e.g. date of onset, date resolved, worst grade, related drug (if known)) - clinician-reported lipodystrophy - lipids (cholesterol, triglycerides, HDL/LDL)) |
| Hepatitis B & C co-infection | - Details of test results |
| Hospitalisation | - details of any inpatient stays (e.g., name of hospital, type of ward, date of admission, date discharged, diagnosis) |
| Female only questions | - date of onset of menarche, pregnancy details (if pregnant, outcome, date of birth of infant if live birth) |
| Patient status | - transferred to another clinic (paediatric/adolescent or adult clinic); known to have left the country; lost to follow up; died (if so separate death form to complete) |
