# Supplementary material for: Factors associated with engagement in HIV care for young people living with perinatally acquired HIV in England: An exploratory observational cohort study
Source: PLoS One. 2024 May 24;19(5):e0302601. doi: 10.1371/journal.pone.0302601 (PMC11125550; doi:10.1371/journal.pone.0302601)
Supplement: S2 File — (DOCX) [file pone.0302601.s005.docx]

S2 File. Variables included in the analysis and rationale for inclusion

S2 Table: Variables included in the analysis and rationale for inclusion, by domain

| *Domain*, exposure variables | Study | Exposure – outcome rationale for inclusion in this analysis (references) |
| --- | --- | --- |
| *A priori* (included in all multivariable models) |  |  |
| Time from AALPHI interview (months) | AALPHI | To understand the effect of time since AALPHI variables were collected |
| Sex | AALPHI | Males associated with lower EIC (1,2) |
| Age at study entry | AALPHI | Older child age associated with lower EIC (1–4)  Adolescents have lower EIC compared to adults (5–9) |
| Ethnicity | AALPHI | Ethnicity associated with EIC but varying trends across studies (1,2,6–8,10–12) |
| Born outside of the UK/Ireland | AALPHI | Being born outside USA associated with higher EIC in USA (1) |
| Sociodemographic |  |  |
| Education/employment status | AALPHI | Unemployment associated with lower EIC (13)  Employment associated with lower EIC (14)  HIV and socioeconomic disadvantage associated with underachievement and mental health problems (15–17) |
| Ever excluded from school | AALPHI | Exclusion from school associated with mental health problems and social exclusion (18,19) |
| Death of parents | AALPHI | Death of parents associated with mental health problems and reduced social support (20–22) |
| Fostered/adopted | AALPHI | People who were orphaned and have frequent carers had lower EIC (23) |
| Number of main carers | AALPHI |  |
| Live with parents/carers | AALPHI | Living in a household with another person living with HIV associated with increased EIC (13) |
| Parent/carer in work | AALPHI | Employment associated with lower EIC (14) |
| Main language spoken at home | AALPHI | Speaking a language other than English at home associated with mental health problems (22) |
| Income deprivation affecting children index (IDACI) deprivation score | AALPHI | Low socioeconomic areas associated with increased EIC (2) |
| Risk behaviour practices |  |  |
| Ever smoked cigarettes | AALPHI | Based on expert opinion |
| Had alcohol in the last year | AALPHI | Alcohol misuse associated with lower EIC (7) |
| Alcohol amount | AALPHI | Alcohol misuse associated with lower EIC (7) |
| Ever used recreational drugs | AALPHI | Use of recreational drugs (ever) associated with mental health problems (24) and lower EIC (1,11,24,25,26) |
| Ever had sex (vaginal or anal) | AALPHI | Sexual activity in young people associated with mental health problems (27) |
| Age at first sex | AALPHI | Pre-adult sexual activity associated with mental health problems (24) |
| Condom use | AALPHI | Based on expert opinion |
| Mental health |  |  |
| Feelings about HIV | AALPHI | Acceptance of HIV associated with increased EIC (3) |
| Ever self-harmed | AALPHI | Self-harm associated with other mental health problems (28) |
| Ever felt life was not worth living | AALPHI | Ever felt life was not worth living is associated with other mental health problems (22) |
| Major life events | AALPHI | Negative life events cause other mental health problems (29,30) |
| Pediatric Quality of Life Inventory (PedsQL^TM^) | AALPHI | Lower social functioning associated with other mental health problems (18) |
| Rosenberg Self-Esteem Scale | AALPHI | Self-esteem associated with other mental health problems (18,28) |
| Hospital anxiety and depression Scale (HADS) | AALPHI | Anxiety associated with other mental health and behavioural problems (31) |
|  |  | Depression associated with lower EIC (7) |
| Cognition domain |  |  |
| Neuropsychological test composite z score-6 (NPZ-6) score^1^ | AALPHI | Cognitive impairment associated with worse EIC (7)  Poorer cognition associated with depression (32) |
| Clinic |  |  |
| Clinic location | CHIPS | Based on expert opinion |
| Clinic type | CHIPS | Adolescent friendly services associated with better EIC (33–37) |
| Distance from home to clinic (km) | CHIPS & AALPHI | Further distance from home to clinic associated with worse EIC (38) |
| Travel time from home to clinic (min) | CHIPS & AALPHI | Longer travel time from home to clinic associated with worse EIC (38) |
| HIV experience and management |  |  |
| Age told HIV diagnosis | AALPHI | Had experience of disclosure associated with lower EIC (23) |
| Number of people told about HIV | AALPHI | Difficulty disclosing to others associated with increased internalised stigma and social isolation and decreased EIC (39) |
| Doses of missed in last 3 days | AALPHI | Missed doses associated with mental health problems (24) |
| Self-assessment of adherence | AALPHI | Associated with mental health problems (24) |
| HIV markers |  |  |
| HIV severity: |  |  |
| Previous CDC C event | CHIPS | Previous AIDS diagnosis associated with higher EIC (40)  Lower EIC associated with CDC C events (41) |
| Nadir CD4 cell count (cells/µL ) | CHIPS | Higher CD4 nadir associated higher EIC (42) |
| CD4 cell count (cells/µL ) | CHIPS | Lower CD4 count associated with lower EIC (7,40,43) |
| Viral load (c/mL) | CHIPS | Treatment failure before disengagement associated with lower EIC (9,35,40,41,43–45) |
| ART: |  |  |
| Age at ART start | CHIPS | Younger age at ART start associated with lower EIC (44) |
| On efavirenz | CHIPS | Efavirenz associated with depression (46) |
| Treatment interruption ≥30 days | CHIPS | Taking ART associated with better EIC (43,47) |

**^1^** Summary score of six cognition domains

**References**

1. Gray KM, Bañez Ocfemia MC, Wang X, Li J, Nesheim SR. *Characteristics and care outcomes among persons living with perinatally acquired HIV infection in the United States, 2015*. JAIDS Journal of Acquired Immune Deficiency Syndromes. 2019;82(1):1.

2. Gebrezgi MT, Sheehan DM, Mauck DE, Fennie KP, Ibanez GE, Spencer EC, et al. *Individual and neighborhood predictors of retention in care and viral suppression among Florida youth (aged 13–24) living with HIV in 2015*. Int J STD AIDS. 2019;30(11):1095–104.

3. Philbin MM, Tanner AE, DuVal A, Ellen JM, Kapogiannis B, Dennis Fortenberry J. *Understanding care linkage and engagement across 15 adolescent clinics: Provider perspectives and implications for newly HIV-infected youth*. AIDS Education and Prevention. 2017;29(2):93–104.

4. Kranzer K, Bradley J, Musaazi J, Nyathi M, Gunguwo H, Ndebele W, et al. *Loss to follow-up among children and adolescents growing up with HIV infection: age really matters*. Journal of the International AIDS Society. 2017;20(1):1–7.

5. Enane LA, Vreeman RC, Foster C. *Retention and adherence: global challenges for the long-term care of adolescents and young adults living with HIV*. Curr Opin HIV AIDS. 2018;1.

6. Farmer C, Yehia BR, Fleishman JA, Rutstein R, Mathews WC, Nijhawan A, et al. *Factors Associated With Retention Among Non-Perinatally HIV-Infected Youth in the HIV Research Network*. Journal of the Pediatric Infectious Diseases Society. 2014;5(1):1–8.

7. Howarth A, Apea V, Michie S, Morris S, Sachikonye M, Mercer C, et al. *REACH: a mixed-methods study to investigate the measurement, prediction and improvement of retention and engagement in outpatient HIV care*. Health Services and Delivery Research. 2017;5(13):1–160.

8. Horberg MA, Hurley LB, Silverberg MJ, Klein DB, Quesenberry CP, Mugavero MJ. *Missed office visits and risk of mortality among hiv-infected subjects in a large healthcare system in the United States*. AIDS Patient Care STDS. 2013;27(8):442–9.

9. Doshi RK, Milberg J, Isenberg D, Matthews T, Malitz F, Matosky M, et al. *High Rates of retention and viral suppression in the US HIV safety net system: HIV care continuum in the ryan white HIV/AIDS program, 2011*. Clinical Infectious Diseases. 2015;60(1):117–25.

10. Dasgupta S, Oster AM, Li J, Hall I. *Disparities in Consistent Retention in HIV Care — 11 States and the District of Columbia, 2011–2013*. Centers for Disease Control and Prevention Morbidity and Mortality Weekly Report. 2016;65(4):77–82.

11. Hall, H.I., K.M. G, T. T, J. L, L. S. *Retention in care of adults and adolescents living with HIV in 13 US areas*. Journal of Acquired Immune Deficiency Syndromes. 2012;60(1):77–82.

12. Israelski D, Gore-Felton C, Power R, Wood MJ, Koopman C. *Sociodemographic characteristics associated with medical appointment adherence among HIV-seropositive patients seeking treatment in a county outpatient facility*. Preventive Medicine. 2001;33(5):470–5.

13. Brown LB, Ayieko J, Mwangwa F, Owaraganise A, Kwarisiima D, Jain V, et al. *Predictors of retention in HIV care among youth (15-24) in a universal test-and-treat setting in rural Kenya*. Journal of Acquired Immune Deficiency Syndromes. 2017;76(1):e15–8.

14. Hussen SA, Harper GW, Bauermeister JA, Hightow-Weidman LB. *Psychosocial influences on engagement in care among HIV-positive young black gay/bisexual and other men who have sex with men.* AIDS patient care and STDs. 2015;29(2):77–85.

15. Stokes L, Rolfe H, Hudson- N. *A compendium of evidence on ethnic minority resilience to the effects of deprivation on attainment*. 2015;(June).

16. Hair NL, Hanson JL, Wolfe BL, Pollak SD. *Association of Child Poverty, Brain Development, and Academic Achievement*. JAMA Pediatrics. 2015;53706(9):1–8.

17. Garvie PA, Zeldow B, Malee K, Nichols SL, Smith RA, Wilkins ML, et al. *Discordance of cognitive and academic achievement outcomes in youth with perinatal HIV exposure.* The Pediatric infectious disease journal. 2014 Sep;33(9):e232-8.

18. le Prevost M, Melvin D, Nunn A, Arenas-Pinto A, Evangeli M, Foster C, et al. *Anxiety and depression in young people with perinatal HIV and their HIV-negative siblings*. In: 7th International Workshop on HIV in Pediatrics. Vancouver; 2015.

19. Berridge D, Brodie I, Pitts J, Porteous D, Tarling R. *The independent effects of permanent exclusion from school on the offending careers of young people*. RDS Occasional Paper. 2001;(71):i–58.

20. Battles HB, Wiener LS. *From adolescence through young adulthood: psychosocial adjustment associated with long-term survival of HIV*. J Adolesc Health. 2002;30(3):161–8.

21. Lewis J V, Abramowitz S, Koenig LJ, Chandwani S, Orban L. *Negative life events and depression in adolescents with HIV: a stress and coping analysis.* AIDS care. 2015 Oct;27(10):1265–74.

22. Le Prevost M, Arenas-Pinto A, Melvin D, Parrott F, Foster C, Ford D, et al. *Anxiety and depression symptoms in young people with perinatally acquired HIV and HIV affected young people in England*. AIDS Care. 2018;30(8):1040–9.

23. Zanoni BC, Archary M, Subramony T, Sibaya T, Psaros C, Haberer JE. *Disclosure, Social Support, and Mental Health are Modifiable Factors Affecting Engagement in Care of Perinatally-HIV Infected Adolescents: A Qualitative Dyadic Analysis*. AIDS Behav. 2021;25(1):237–48.

24. Kapetanovic S, Wiegand RE, Dominguez K, Blumberg D, Bohannon B, Wheeling J, et al. *Associations of Medically Documented Psychiatric Diagnoses and Risky Health Behaviors in Highly Active Antiretroviral Therapy-Experienced Perinatally HIV-Infected Youth*. AIDS Patient Care and STDs. 2011;25(8):493–501.

25. Dietz BA, Clum GA, Chung S, Leonard L, Murphy DA, Perez L V, et al. *Adherence to Scheduled Appointments among HIV-Infected Female Youth in Five US Cities*. J Adolesc Health. 2010;46(3):278–83.

26. Gwadz M, de Guzman R, Freeman R, Kutnick A, Silverman E, Leonard NR, et al. *Exploring How Substance Use Impedes Engagement along the HIV Care Continuum: A Qualitative Study*. Frontiers in Public Health. 2016;4(April).

27. Judd A, Foster C, Thompson LC, Sturgeon K, Le Prevost M, Jungmann E, et al. *Sexual health of young people with perinatal HIV and HIV negative young people in England*. PLoS One. 2018;13(10):e0205597.

28. Copelyn J, Thompson LC, Prevost M Le, Castro H, Sturgeon K, Rowson K, et al. *Self-harm in young people with perinatal HIV and HIV negative young people in England: cross sectional analysis.* BMC Public Health. 2019 Aug;19(1):1165.

29. Mutumba M, Bauermeister JA, Harper GW, Musiime V, Lepkowski J, Resnicow K, et al. *Psychological distress among Ugandan adolescents living with HIV: Examining stressors and the buffering role of general and religious coping strategies.* Global public health. 2016 Apr 11;1–13.

30. Gosling AS, Burns J, Hirst F. *Children with HIV in the UK: A Longitudinal Study of Adaptive and Cognitive Functioning*. Clinical Child Psychology and Psychiatry. 2004;9(1):25–37.

31. Mellins CA, Malee KM. *Understanding the mental health of youth living with perinatal HIV infection: lessons learned and current challenges.* J Int AIDS Soc. 2013 Jan;16(1):18593.

32. Judd A, le Prevost M, Melvin D, Arenas-Pinto A, Parrott F, Winston A, et al. *Cognitive function in young people with and without perinatal HIV in the AALPHI cohort in England: the role of non-HIV related factors.* Clinical infectious diseases. 2016 Aug 31;63(10):1380–7.

33. Teasdale C, Alwar T, Chege D, Hawken M, Abrams E. *Impact of Youth and Adolescent Friendly Services on Retention of 10–24-Year-Olds in HIV Care and Treatment Programs in Nyanza, Kenya*. Journal of the International AIDS Society. 2015;56–9.

34. Davila J a., Miertschin N, Sansgiry S, Schwarzwald H, Henley C, Giordano TP. *Centralization of HIV services in HIV-positive African-American and Hispanic youth improves retention in care*. AIDS Care. 2012;0121(May 2015):1–5.

35. Judd A, Sohn AH, Collins IJ. *Interventions to improve treatment, retention and survival outcomes for adolescents with perinatal HIV-1 transitioning to adult care: moving on up.* Current opinion in HIV and AIDS. 2016;11(5):477–86.

36. Lee L, Yehia BR, Gaur AH, Rutstein R, Gebo K, Keruly JC, et al. *The Impact of Youth-Friendly Structures of Care on Retention Among HIV-Infected Youth*. AIDS Patient Care STDS. 2016;30(4):170–7.

37. Fortenberry JD, Koenig LJ, Kapogiannis BG, Jeffries CL, Ellen JM, Wilson CM. *Implementation of an integrated approach to the national HIV/AIDS strategy for improving human immunodeficiency virus care for youths*. JAMA Pediatrics. 2017;171(7):687–93.

38. Ridgway JP, Almirol EA, Schmitt J, Schuble T, Schneider JA. *Travel Time to Clinic but not Neighborhood Crime Rate is Associated with Retention in Care Among HIV ‑ Positive Patients*. AIDS and Behavior. 2018;22(9):3003–8.

39. Zanoni BC, Sibaya T, Cairns C, Haberer JE. *Barriers to Retention in Care are Overcome by Adolescent-Friendly Services for Adolescents Living with HIV in South Africa: A Qualitative Analysis*. AIDS Behav. 2018;23(4):957–65.

40. Gingaras C, Smith C, Radoi R, Sima D, Youle M, Ene L. *Engagement in care among youth living with parenterally-acquired HIV infection in Romania*. AIDS Care - Psychological and Socio-Medical Aspects of AIDS/HIV. 2019;31(10):1290–6.

41. Cuzin L, Dellamonica P, Yazdanpanah Y, Bouchez S, Rey D, Hoen B, et al. *Characteristics and consequences of medical care interruptions in HIV-infected patients in France*. Epidemiology and Infection. 2016;144(11):2363–70.

42. Howarth A, Burns F, Apea V, Jose S, Hill T, Delpech V, et al. *Development and application of a new measure of engagement in out-patient HIV care*. HIV Medicine. 2016;1–8.

43. Berg MB, Safren SA, Mimiaga MJ, Grasso C, Boswell S, Mayer KH. *Nonadherence to medical appointments is associated with increased plasma HIV RNA and decreased CD4 cell counts in a community-based HIV primary care clinic*. AIDS Care - Psychological and Socio-Medical Aspects of AIDS/HIV. 2005;17(7):902–7.

44. Bartlett AW, Lumbiganon P, Jamal Mohamed TA, Lapphra K, Muktiarti D, Du QT, et al. *Dual Analysis of Loss to Follow-up for Perinatally HIV-Infected Adolescents Receiving Combination Antiretroviral Therapy in Asia*. Journal of Acquired Immune Deficiency Syndromes. 2019;82(5):431–8.

45. Pantelic M, Casale M, Cluver L, Toska E, Moshabela M. *Multiple forms of discrimination and internalized stigma compromise retention in HIV care among adolescents: findings from a South African cohort*. J Int AIDS Soc. 2020;23(5):1–8.

46. Gaida R, Truter I, Grobler C. *Efavirenz: A review of the epidemiology, severity and management of neuropsychiatric side-effects*. South African Journal of Psychiatry. 2015;21(3):94–7.

47. Carlucci JG, Liu Y, Clouse K, Vermund SH. *Attrition of HIV-positive children from HIV services in low-A nd middle-income countries: A systematic review and meta-analysis*. Aids. 2019;(July 2019).
