# Supplementary material for: Factors associated with engagement in HIV care for young people living with perinatally acquired HIV in England: An exploratory observational cohort study
Source: PLoS One. 2024 May 24;19(5):e0302601. doi: 10.1371/journal.pone.0302601 (PMC11125550; doi:10.1371/journal.pone.0302601)
Supplement: S3 File — (DOCX) [file pone.0302601.s006.docx]

# S3 File. Early attendance for appointments

## Rationale and methods

During the analysis, patients who attended their visits were categorised as either on time, or early (both of which would be considered in care) or late (out of care). However if too many participants were classified as attending visits early, this could indicate that parts of the flowcharts are not representative of clinical practice. To check how frequently this occurred a variable was created describing the actual number of months between visits, and compared to the scheduled next appointment time frame. The last appointment for all participants within the 12 month period was dropped because it was not possible to assess if participants attended early or not for this appointment.

All participants were then classified as ‘not early’ (which included both participants who attended on time and those who attended late) or ‘early’. Participants cannot come early to a scheduled appointment classified as one month, so scheduled appointments of 1 month were not examined. To allow for the reality of booking appointments in busy NHS clinics, some leniency was added into the time frames, so that any participant attending one month early for a visit was considered ‘not early’. For this reason, time to next scheduled appointments of 2 months were also not examined any further.

Two analyses were undertaken to investigate the potential effect of early attenders for 6, 4 or 3 month appointments on the results of my analyses. Firstly, early attenders were investigated to see if they were different in characteristics to those who attended on time or late. If early attenders were different in some way, this might point to criteria in the flowchart being wrong and the need for additional decision boxes to incorporate the characteristic which was different. Therefore, key characteristics were compared (e.g. age group, sex, ethnicity, clinic type, on adult doses) of participants who attended early to those who were not early. Definitions of flowchart classifications were also altered (e.g. CD4 groupings, consecutive CD4 drop to single CD4 change, viral load log increase from 0.5 log to 1.0 and 0.25 log changes) in the flowchart to see if this highlighted a difference between participants who attended early vs. not early to their visit. Secondly, to understand the effect of early attenders in more detail, the actual time to the next visit was then cross tabulated with all the possible terminal nodes for each of the three EIC flowcharts. If the proportion of early visits out of the total visits in each terminal node was a gross departure (e.g. more than half of the participants came early) it might indicate that the scheduling for the next appointment for that terminal node was not reflective of actual clinical practice, and might need to be shortened.

## Results

#### Comparison or participants attending not early vs. early

S1 Table shows the number of visits that participants attended earlier than their next scheduled appointment date as well as a summary of months attended early or not. A total of 262/678 (39%,) visit months were earlier than originally scheduled. Around a third of participants attended early for next scheduled appointments at 3 (35%, 65/185) or 4 (29%, 112/383) months’ time. Three quarters of participants (77%, 85/110) whose time to next scheduled appointment was in 6 months’ time, visits were early, with most participants attending 2 (23%) or 3 (30%) months early.

S3 Table 1. Number of visits attended early by time frame of next scheduled appointments

|  | **Time to next scheduled appointment: 3 months (n=185)** | | | **Time to next scheduled appointment: 4 months (n=383)** | | | | **Time to next scheduled appointment: 6 months (n=110)** | | | | | | **Total(n=678)** | | | | | | |
| --- | --- | --- | --- | --- | --- | --- | --- | --- | --- | --- | --- | --- | --- | --- | --- | --- | --- | --- | --- | --- |
|  | n (%) | | | | | | | | | | | | | | | | | | | |
| # of early or not early visits | Not early | | Early | Not early | | Early | | Not early | | Early | | | | Not early | | Early | | | | |
|  | 120 (65%) | | 65 (35%) | 271 (71%) | | 112 (29%) | | 25 (23%) | | 85 (77%) | | | | 416 (61%) | | 262 (39%) | | | | |
| Timing of early visits | Not early | 1m* early | 2m early | Not early | 1m early | 2m early | 3m early | Not early | 1m early | 2m early | 3m early | 4m early | 5m early | Not early | 1m early | | 2m early | 3m early | 4m early | 5m early |
|  | 77 (42%) | 43 (23%) | 65 (35%) | 131 (34%) | 140 (36%) | 60 (16%) | 52 (14%) | 8 (7%) | 17 (15%) | 25 (23%) | 32 (30%) | 11 (10%) | 17 (15%) | 216 (32%) | 200 (29%) | | 150 (22%) | 84 (12%) | 11 (2%) | 17 (3%) |

*m=month/s

The comparisons across appointment schedules should be viewed with caution because there was greater opportunity for participants to be early for a six month appointment than a 3 or 4 month appointment.

Characteristics of participants attending not early vs. early

Key characteristics and slightly altered definitions of flowchart classifications were compared for participants who attended on time (not early) and participants attending before the scheduled appointment dates (early) at 3, 4, and 6 month appointments. Results are presented in S2 Table, S3 Table and S4 Table and no major differences were found.

S3 Table 2. Key characteristics of participants attending not early vs. early for their 3-month appointment

|  | Not early for 3 month appointment n=120 (%) | | Early for 3 month appointment n=65 (%) | | p value |
| --- | --- | --- | --- | --- | --- |
| Participant characteristic |  |  |  |  |  |
| Age group |  |  |  |  |  |
| ≤15 years of age | 47 | (39%) | 24 | (37%) | 0.93 |
| 16-18 years of age | 52 | (43%) | 30 | (46%) |  |
| 19-21 years of age | 21 | (18%) | 11 | (17%) |  |
| Sex |  |  |  |  |  |
| Male | 48 | (40%) | 35 | (54%) | 0.07 |
| Female | 72 | (60%) | 30 | (46%) |  |
| Ethnicity |  |  |  |  |  |
| Black | 105 | (87%) | 59 | (91%) | 0.54 |
| Asian/mixed/prefer not to say | 13 | (11%) | 6 | (9%) |  |
| White | 2 | (2%) | 0 | (0%) |  |
| On all adult ART doses |  |  |  |  |  |
| Yes | 146 | (54%) | 50 | (45%) | 0.08 |
| No | 114 | (42%) | 58 | (52%) |  |
| Missing | 10 | (4%) | 4 | (3%) |  |
| Re-categorised flowchart definitions |  |  |  |  |  |
| CD4 category^1^ |  |  |  |  |  |
| ≥600 cells/μL | 24 | (20%) | 11 | (17%) | 0.25 |
| 500-599 cells/μL | 13 | (11%) | 5 | (8%) |  |
| 400-499 cells/μL | 19 | (24%) | 10 | (15%) |  |
| <400 cells/μL | 54 | (45%) | 39 | (60%) |  |
| CD4 change |  |  |  |  |  |
| ≥50 cells/μL lower than previous | 37 | (31%) | 12 | (19%) | 0.10 |
| Same as previous | 69 | (59%) | 47 | (75%) |  |
| ≥50 cells/μL higher than previous | 12 | (10%) | 4 | (6%) |  |
| VL change |  |  |  |  |  |
| >1.0 log lower than previous | 41 | (35%) | 22 | (65%) |  |
| Same as previous | 74 | (42%) | 41 | (35%) | 0.31 |
| >1.0 log higher than previous | 4 | (3%) | 0 | (0%) |  |
| VL change |  |  |  |  |  |
| >0.25 log lower than previous | 55 | (46%) | 30 | (48%) | 0.95 |
| Same as previous | 55 | (46%) | 29 | (46%) |  |
| >0.25 log higher than previous | 9 | (8%) | 4 | (6%) |  |

^1^ CD4 cell count is categorised into a greater number of categories than in the main analyses

S3 Table 3. Key characteristics of participants attending not early vs. early for their 4-month appointment

|  | Not early for 4 month appointment n=271 (%) | | Early for 4 month appointment n=112 (%) | | p value | |  |
| --- | --- | --- | --- | --- | --- | --- | --- |
| Participant characteristic | | | | | | |  |
| Age group |  |  |  |  |  | |  |
| ≤15 years of age | 163 | (60%) | 70 | (62%) | 0.42 | |  |
| 16-18 years of age | 92 | (34%) | 39 | (35%) |  |  |  |
| 19-21 years of age | 16 | (6%) | 3 | (3%) |  | |  |
| Sex |  |  |  |  |  | |  |
| Male | 111 | (41%) | 48 | (43%) | 0.73 | |  |
| Female | 160 | (59%) | 64 | (57%) |  |  |  |
| Ethnicity |  |  |  |  |  | |  |
| Black | 232 | (86%) | 99 | (88%) | 0.76 | |  |
| Asian/mixed/prefer not to say | 28 | (10%) | 9 | (8%) |  |  |  |
| White | 11 | (4%) | 4 | (4%) |  |  |  |
| On all adult ART doses |  |  |  |  |  | |  |
| Yes | 146 | (54%) | 50 | (45%) | 0.08 | |  |
| No | 114 | (42%) | 58 | (52%) |  |  |  |
| Missing | 10 | (4%) | 4 | (3%) |  | |  |
| Re-categorised flowchart definitions | | | | | | | |
| CD4 category^1^ |  |  |  |  |  | |  |
| ≥600 cells/μL | 175 | (64%) | 74 | (66%) | 0.84 | |  |
| 500-599 cells/μL | 51 | (19%) | 17 | (15%) |  |  |  |
| 400-499 cells/μL | 34 | (13%) | 15 | (14%) |  |  |  |
| <400 cells/μL | 12 | (4%) | 6 | (5%) |  |  |  |
| CD4 change |  |  |  |  |  | |  |
| ≥50 cells/μL lower than previous | 75 | (29%) | 26 | (24%) | 0.15 | |  |
| Same as previous | 112 | (43%) | 59 | (54%) |  |  |  |
| ≥50 cells/μL higher than previous | 73 | (28%) | 24 | (22%) |  |  |  |
| VL change |  |  |  |  |  | |  |
| >1 log lower than previous | 15 | (6%) | 11 | (10%) |  | |  |
| Same as previous | 243 | (93%) | 98 | (88%) | 0.31 | |  |
| >1 log higher than previous | 3 | (1%) | 2 | (2%) |  | |  |
| VL change |  |  |  |  |  | |  |
| >0.25 log lower than previous | 31 | (16%) | 18 | (16%) | 0.53 | |  |
| Same as previous | 218 | (79%) | 88 | (79%) |  |  |  |
| >0.25 log higher than previous | 12 | (5%) | 5 | (5%) |  | |  |

^1^ CD4 cell count is categorised into a greater number of categories than in the main analyses

S3 Table 4. Key characteristics of participants attending not early vs. early for their 6-month appointment

|  | Not early for  6 month appointment  n=25 (%) | | Early for  6 month appointment  n=85 (%) | | | p value | | |  |  |
| --- | --- | --- | --- | --- | --- | --- | --- | --- | --- | --- |
| Participant characteristic |  |  |  |  |  |  |  |  |  |  |
| Age group |  |  |  | |  | | |  | | |
| ≤15 years of age | 1 | (4%) | 1 | | (1%) | | | 0.50 | | |
| 16-18 years of age | 8 | (32%) | 35 | | (41%) | | |  |  |  |
| 19-21 years of age | 16 | (64%) | 49 | | (58%) | | |  | | |
| Sex |  |  |  | |  | | |  | | |
| Male | 12 | (48%) | 33 | | (39%) | | | 0.41 | | |
| Female | 13 | (52%) | 52 | | (61%) | | |  |  |  |
| Ethnicity |  |  |  | |  | | |  | | |
| Black | 24 | (96%) | 73 | | (86%) | | | 0.27 | | |
| Asian/mixed/prefer not to say | 0 | (0%) | 8 | | (9%) | | |  |  |  |
| White | 1 | (4%) | 4 | | (5%) | | |  |  |  |
| Clinic type |  |  |  | |  | | |  | | |
| Adolescent | 17 | (68%) | 60 | | (71%) | | | 0.18 | | |
| Adult/GUM | 6 | (26%) | 24 | | (28%) | | |  |  |  |
| On all adult ART doses |  |  |  | |  | | |  | | |
| Yes | 13 | (52%) | 39 | | (46%) | | | 0.59 | | |
| No | 12 | (48%) | 46 | | (54%) | | |  |  |  |
| Re-categorised flowchart definitions |  | | |  | | |  | | |  |
| CD4 category^1^ |  |  |  | |  | | |  | | |
| ≥600 cells/μL | 14 | (56%) | 60 | | (71%) | | | 0.14 | | |
| 500-599 cells/μL | 7 | (28%) | 9 | | (11%) | | |  | | |
| 400-499 cells/μL | 3 | (12%) | 8 | | (9%) | | |  | | |
| <400 cells/μL | 1 | (4%) | 8 | | (9%) | | |  | | |

^1^ CD4 cell count is categorised into a greater number of categories than in the main analyses

Appointments attended early by terminal node

In total, 38% (n=195) of terminal nodes where participants attended early fell in Group A Flowchart S5 Table). The terminal node where appointments were most frequently attended early was for young people with a CD4 count >350 in adult care. This raised a question about whether it was common practice outside of the clinicians interviewed for this analysis to schedule clinic appointments for young people for 6 months. Unfortunately, there was not time to interview a wider range of clinicians; instead a sensitivity analysis was conducted in which the maximum time to next scheduled appointment was set at 4 months (instead of 6). The results are described in Appendix 5.

S3 Table 5. Summary of appointments attended early by terminal node – Group A Flowchart (on ART viral load ≤50c/mL)

| Flowchart classification details^1^ | On cART | | | | | | | On 1 or 2 drugs | | | All terminal nodes |
| --- | --- | --- | --- | --- | --- | --- | --- | --- | --- | --- | --- |
|  | CD4 ≤200 for | CD4 201-350 | | | CD4 >350 | | | ≤1 year | >1 year | |  |
|  | >1 year | ≤1 year | >1 year | |  | | |  |  |  |  |
| Terminal node details^2^ | Weight ≥40kg or on adult doses |  | In adult care, Weight ≥40kg or on adult doses | In paed care, Weight ≥40kg or on adult doses | In adult care, Weight ≥40kg or on adult doses | In paed care, Weight ≥40kg or on adult doses | Weight <40kg (in adult or paed care) & not on adult doses |  | In adult care, Weight ≥40kg or on adult doses | In paed care, Weight ≥40kg or on adult doses |  |
| Visit frequency^3^ | 4 months | 3 months | 6 months | 4 months | 6 months | 4 months | 3 months | 3 months | 6 months | 4 months |  |
| Number of early visits/ total visits (%) | 1/1^4^(100%) | 7/23 (30%) | 1/1 (100%) | 2/5 (40%) | 78/103 (76%) | 93/330 (28%) | 0/7 (0%) | 2/17 (12%) | 6/6 (100%) | 5/18 (28%) | 195/511 (38%) |

^1^ None of the participants in this table had new ART at that clinic visit

^2^ Terminal nodes in which no participants attended the clinic appointment early are not shown in the table

^3^ As above due to a month’s leniency being added to early appointments, participants cannot be classified as early for visits with a 1 or 2 month follow-up time frame. Therefore, only visits with scheduled appointment time for 3, 4 or 6 months are included in this summary table.

^4^ Total months differ from those in Figures 3.8-3.10 due to last visit being dropped for each participant

Forty-seven per cent of early visits were in Group B Flowchart (S6 Table). These early visits were spread across four terminal nodes. Early attenders fell most commonly in the terminal node where participants had a CD4 count ≤250 cells/μL, with an increase, no change or drop of <50 in CD4 since last visit. Although the proportion is just over 50%, the numbers are quite small and it is perhaps not that surprising as this is one of the three terminal nodes with most visits in Group B Flowchart.

S3 Table 6. Summary of appointments attended early by terminal node – Group B Flowchart (on ART viral load >50c/mL)

| Flowchart classification details | On ART> 6 months, on PI, previous VL>50, VL decrease or no change | | | | All terminal  nodes |
| --- | --- | --- | --- | --- | --- |
|  | CD4 ≤350, | CD4 351-499 | | CD4 ≥500 |  |
| Terminal node details | Increase/no change or drop <50 in CD4 since last visit | Increase/no change or drop <50 in CD4 since last visit | First drop ≥50 in CD4 since last visit |  |  |
| Visit frequency^1^ | 3 months | 3 months | 3 months | 3 months |  |
| Number of early visits / total visits (%) | 26/46 (57%) | 11/28 (39%) | 1/4 (25%) | 14/32 (44%) | 52/110 (47%) |

^1^ Due to a month’s leniency being added to early appointments, participants cannot be classified as early for visits with a 1 or 2 month follow-up time frame. Therefore, visits with scheduled appointment time for 3, 4 or 6 months are included in this summary table.

Finally, 26% (n= 15) of the early visits fell in Group C Flowchart (S7 Table). These early visits were spread across three terminal nodes and the numbers of visits were relatively small.

S3 Table 7. Summary of appointments attended early by terminal node - Group C Flowchart (not on ART)

| Flowchart classification details | No CDC C in last 3 months | | | All terminal nodes |
| --- | --- | --- | --- | --- |
|  | CD4 351-499 | | CD4 ≥500 |  |
| Terminal node details | Increase/ no change or drop <50 in CD4 since last visit | Single drop ≥50 in CD4 since last visit |  |  |
| Visit frequency1 | 3 months | 3 months | 4 months |  |
| Number of early visits / total visits (%)1 | 3/23 (13%) | 1/5 (20%) | 11/29 (38%) | 15/57 (26%) |

^1^ Due to a month’s leniency being added to early appointments, participants cannot be classified as early for visits with a 1 or 2 month follow-up time frame. Therefore, visits with scheduled appointment time for 3, 4 or 6 months are included in this summary table.
